# The ERC *PuppetPlays* project : contribution for a non-linear history of the European theatre

**DOI:** 10.12688/openreseurope.15807.1

**Published:** 2023-05-02

**Authors:** Didier Plassard

**Affiliations:** 1 Théâtre et spectacle vivant, Universite Paul-Valery Montpellier 3, Montpellier, Occitanie, 34090, France

**Keywords:** Puppetry, Theatre History, Drama, Performing Arts

## Abstract

This article is a presentation of the ERC Advanced Grant project *PuppetPlays - Reappraising Western European Repertoires for Puppet and Marionette Theatre (GA 835193)*. After a short overview of the project itself, it begins with a definition of puppetry, based on the phenomenon of double vision. Then it explains the choice of the corpus limitations, describes the variety of the available resources, and underlines the great discrepancy in the amount of material available in the different countries.

The article continues with a brief overview of the role played by puppetry in the wider frame of performing arts: how much can we consider that puppeteers developed specific repertoires? What kind of differences can be observed between puppet or marionette theatre and actors’ theatre? The answers to these questions differ in a considerable way according to the cultural and sociological contexts: sometimes puppet and marionette theatre were the only forms of performance allowed, and they acted as substitutes for actors theatre; but sometimes also - and this is increasingly the case since the end of the 19th century -  these instruments were chosen for their specific expressive qualities.

In a last movement, I emphasize that collecting and analyzing puppet and marionette repertoires brings us to reconsider the general historiography of theatre: firstly, because we bring into the light theatrical genres that have been neglected by the historians; and secondly, because the plays written by the puppeteers, when we look closely at them, reveal a stratification of different layers that can be considered as a kind of heterochrony ; an alternative construction to social time. The forgotten patrimony of puppet and marionette dramaturgy conceals therefore many possibilities for research in humanities and social sciences.

## Plain language summary

This article is a presentation of the ERC Advanced Grant project, *PuppetPlays - Reappraising Western European Repertoires for Puppet and Marionette Theatres*. After a short overview of the project itself, it begins with a definition of puppetry, then it explains the choice of the corpus limitations, describes the variety of the available resources, and underlines the great discrepancy in the amount of material available in the different countries.

The article continues with a simple question: did puppeteers perform specific repertoires? What kind of differences can be observed between the plays performed by puppeteers and those performed by actors? The answers to these questions are very different according to the circumstances: sometimes puppet and marionette theatre were the only forms of performance allowed, and they acted as substitutes for actors’ theatre; but sometimes also - and this is increasingly the case since the end of the 19th century - these instruments (puppets, marionettes, shadow figurines, etc.) were chosen for their specific expressive qualities, as a tool for experimental performances.

In a last movement, I emphasize that collecting and analyzing the plays for puppet and marionette brings us to reconsider the way theatre history is usually written: firstly, because we bring into the light theatrical genres (religious plays, military dramas, etc.) that have been neglected by the historians; and secondly, because the plays written by the puppeteers, when we look closely at them, reveal multiple layers inherited from different times and traditions. The forgotten patrimony of puppet and marionette dramaturgy conceals therefore many possibilities for research in humanities and social sciences.

## Disclaimer

The views expressed in this article are those of the author(s). Publication in Open Research Europe does not imply endorsement of the European Commission.

## Introduction: a brief presentation of *PuppetPlays*

*PuppetPlays – Reappraising Western European Repertoires for Puppet and Marionette Theatres* is a European research project selected from among the European Research Council Advanced Grant proposals in 2018 and financed by the European Union for a period of five years from 1 ^st^ October 2019 to 30 ^th^ September 2024. It was developed, and is directed by, Didier Plassard, professor in theatre studies at the Université Paul-Valéry Montpellier 3. The object of study in this project is the repertoire of texts written for the puppet theatre from the 17 ^th^ century up until the present day. The results obtained at the end of the project will take the form of a conclusive monograph, two doctoral theses, the publication of the proceedings of two conferences, several scientific articles and an internet platform which will contain, amongst other things, a data base and an anthology of rare or hitherto unpublished texts.

## The puppet theatre and double vision

A semantic precision is required at this point. Within the expression “puppet theatre”, we include all the traditional techniques of stage animation (string puppets, glove puppets, rod puppets, rod marionettes, shadow figures…), but also contemporary techniques (direct manipulation, object theatre, costume puppets…): that is all the different methods of theatrical representation in which a staged object is simultaneously perceived as an object and as a living being, according to the phenomenon of “double vision” as theorized by Steve Tillis. ^1^

But speaking of animated objects instead of puppet or marionette is still an approximation. It is indeed difficult to bring together under a unique appellation all the elements used to create a “double vision”, from the immaterial silhouettes of the shadow theatre to parts of the human body: I am thinking for example of the “nano-choreographies” of Michèle Anne de Mey ^2^ in which the hands of the dancers are perceived as if they were the whole body. This is the reason why defining the puppet theatre by enumerating the theatrical instruments which it makes use of, is practically impossible. It is safer to characterize it using the phenomenon of double vision which makes it possible moreover to include the functioning of the puppet in the more general sphere of theatricality. The puppet theatre is indeed an intensified, densified theatre in which the fictional pact established by mimesis is deepened through recourse to duplication. Already the presence of the living actor, on stage, is perceived in a double way: this man is David Garrick, and he is Hamlet. But the art of puppetry enlarges the gap between what we see in reality and what we see in imagination: this figure made of wood and cloth is an object, but it is also a living being, and it is Hamlet.

### The central hypothesis

The central hypothesis of the *PuppetPlays* project can be summed up in a few words. During certain periods and in certain socio-cultural contexts in Europe the fact that a figure made of wood and cloth represented Hamlet did not pose a problem. The reason for this choice was an external one, for example the actors did not give their performances in this or that distant part of the country, or in this or that poor district; or at this period of the year or in that region, the political or the religious authorities forbade the actors to appear on stage; or, a third possibility, the main theatrical institutions managed to obtain from the political powers of the day a monopoly which deprived other companies of the possibility of performing their theatrical texts with real actors on stage. The puppet theatre seems then to be a substitute for the actors’ theatre in contexts where the latter was forbidden to perform, or limited in its faculty of expression, or more simply where it did not normally go.

However, in the history of European theatre, we can find many other social contexts and many other periods in which these two means of representation, the actors’ theatre and the puppet theatre, existed side by side and aimed at the same audience. This was for example the case in 18 ^th^ century Venice or opera performances in private houses during the carnival festivities, or in the Symbolist and Modernist circles in Paris, Munich or Barcelona between the end of the 19 ^th^ century and the beginning of the 20 ^th^ century. And this co-existence is even more evident in contemporary theatre where actors and puppets play in the same theatres, and sometimes share the same stage in hybrid productions of “visual theatre”. The persistent presence of the puppet theatre in such contexts, both ancient and modern, shows that it cannot be kind of default theatre, or a substitute aimed at audiences which do not have access to “real” theatre (or who do not yet have access to it, like children for example).

Puppets – and this is our hypothesis – inherently possess expressive potential that artists and writers who have worked with them have progressively highlighted. From being a simple substitute for the actor, the puppet has become a theatrical instrument, an instrument which, more and more often is precisely chosen for its qualities. Identifying this expressive potential and examining how it has been defined at different periods and in different artistic contexts by the companies and the dramatic authors who have chosen to use the puppet, is the objective of *PuppetPlays*.

Our research is therefore dramaturgical: we examine and interpret the interaction between word and figure through an analysis of texts written for these instruments – which supposes, above all, the researching of sources. And it is this research that I would like, for a moment, to touch on here.

## Mapping the repertoire of puppet theatres in Western Europe

### How the project was born

The *PuppetPlays* project was born from the conviction that our knowledge of the puppet theatre, both from the historiographical and aesthetic point of view, can only be superficial until we have carefully analysed its dramaturgy. However, in order to carry out these analyses, access to the texts is vital, which means that we first need to identify them, and then seek them out. For this reason, a large part of the work carried out within the framework of the *PuppetPlays* project consists in the building of a data base which presents a broad selection of texts taken from the repertoires of puppet theatres in Western Europe: Great Britain, France, Spain, Portugal, Italy, Switzerland, Austria, Germany, Belgium, Luxemburg and the Netherlands. We are restricting ourselves to these countries because they have, since the 16 ^th^ century, experienced the widespread circulation of companies, characters, techniques, and stories which have built up a homogeneous whole, whereas the rest of Europe developed a specific activity in the field of puppetry only much later, from the 19 ^th^ century onwards – and sometimes taking inspiration from other traditions as for the Greek Karaghiozis derived from the Turkish Karagöz.

For each text, the data base, puppetplays.eu, presents a brief introduction, a summary of the plot, the list of characters, some information concerning the first performance, bibliographical and archival references which will enable the researcher to find the text, a description of theatrical techniques (monologue, metamorphosis, apparition…), and the literary registers employed, the animation techniques used, some thematic key-words and finally hyper-textual cross-references when we know them. Also presented are the authors with their permanent identifiers, the main traditional characters and the techniques of animation. The results of a search, either by request or filters, can be visualized in the form of a list or on a geographical map of Europe – a process which seemed to us the best way to arouse the curiosity of users. We have given preference to simple and intuitive navigation in the hope that all those who are interested in puppets (researchers, artists, students, teachers, librarians, museum curators, spectators…) can easily find their way around all this information.

Our initial ambition was to arrive at a selection of 2,000 descriptive articles. However, as we have moved towards a much more detailed description of the texts than we had originally imagined, I think that we will limit ourselves to about a thousand articles. We have around 500 today, and several dozens of them have already been translated into English, as the website will be available in both French and English.

It is one thing to have the descriptive article in the data base with all the information that it contains, but it is another to have the text itself that users – and this is our hope - will want to read directly for themselves. We will therefore include a link to an electronic copy of the text when it is accessible on one of the big internet portals like Gallica, Google Books, Archive.org, Europeana. We are also preparing an online anthology of 300 unpublished texts which will give access to a selection of rare and significant resources.

### Typology of the resources

Many of the texts written for the puppet theatre have remained unpublished. The material that we have gathered, and that we describe in the data base can be organized schematically according to the following typologies:

1) Printed texts by literary authors (poets, novelists, dramatic authors, etc.)
2) Unpublished texts (manuscripts, typed, electronic) by literary authors
3) Printed texts by stage artists (glove puppets, string puppets, rod puppets, etc.)
4) Unpublished texts (manuscripts, typed, electronic) by stage artists
5) Printed texts without indication of authors (“traditional”)
6) Unpublished texts (manuscripts, typed) without indication of authors (“traditional”)
7) Unpublished texts without a written copy (audio and audio-visual recordings)

It should be added that the category of “printed texts” brings together very different objects, from classical works available in academic editions, to writings published more than a century ago in confidential journals which have become difficult to find, or in books that are out of circulation. Not to mention the notion of “author”, here we find Nobel Prize winners for literature as well as teachers who, in their whole lives, have published only one slim volume of tales for children. A strong characteristic of the puppet theatre is the fact that it also brings to the stage texts by amateur writers, and also by the companies themselves (adaptations, parodies, rewritings of all kinds), sometimes inherited from previous generations and transformed to suit the changing tastes of the public. It is not rare for example to find out that a comedy had been performed in the middle of the 18 ^th^ century, but that we are only able to read it thanks to a hand-written copy produced a hundred and fifty years later. This encourages us not only to rethink the categories of authorship and literary originality, but also the historiographical structures we still use to guide our reflection. I shall return to this point later.

### A contrasting landscape

The *PuppetPlays* project must then make its way through a dense and almost virgin forest of texts in which one might meet the same work under different titles, or the same title might be used for different works. Identifying these texts and following their different transformations presents a number of difficulties: it is necessary to verify that the work was really written for the puppet theatre and that it is not merely a revival of a comedy, or a play for actors, or again a simple adaptation for the stage of a children’s tale or book. In order to better identify the dramaturgical specificities of the puppet theatre, but also to broaden our knowledge of its repertoire, we focus on original works or adaptations that demonstrate a significant rewriting of the hypotext.

However, the main difficulties that we have encountered come from the great disparity in the available resources depending on the languages and countries in our study. Indeed, there are enormous differences in the conservation of the puppet theatre repertoires in Western Europe, as far as the treatment of archives is concerned, or the publication of texts, or the role attributed to authors in the process of creation.

In Italy, for example, we can find very few texts written by literary authors, but on the other hand there is a vast quantity of hand-written texts in the different places where they are stored: libraries, museums, foundations, archives and private collections… Dozens and sometimes hundreds of manuscripts from the 19 ^th^ century and the first half of the 20 ^th^ century are held in many towns. To give just one example, the collection of the puppeteer Peppino Sarina (1884–1978) in the small town of Tortona (Alessandria province) brings together exactly 2,981 manuscripts. Throughout the peninsula the wealth of local traditions and the large number of artistic dynasties (the *famiglie d’arte*) have led to the creation of a huge, but also fragmented patrimony. In this case, our problem resides in the choice of the most interesting or the most significant versions in the midst of the profusion of disseminated sources which are almost always hand-written. So, we match our own selection criteria (age, diversity, variations on the same story) with the advice given by the curators and collectors concerned.

In Germany the collections are centralized in a small number of museums, but there are very many of them: about 1,500 hand-written or typed texts in Munich, as many in Dresden, several hundred in Magdeburg or Cologne. The Germanic zone is characterized especially by the very great number of author texts, ranging from the most important names in literary history (Goethe, Tieck, Eichendorff, von Armin, Hofmannsthal, Hauptmann, Schnitzler, Bernhard, Jelinek…) to the lesser-known names of reformer pedagogues from the different provinces in the first half of the 20 ^th^ century. We find very many published volumes, including those by completely unknown authors for whom it is difficult to piece together a biography, and to know whether their texts were ever performed.

France offers a third type of picture, partly comparable to that of Germany for the number of authors who have written for puppets from the 17 ^th^ century until the present day. Here too great collections have been assembled, particularly in the *Musées Gadagne* in Lyons (which hold 1,451 texts for Guignol) and at the *Institut International de la Marionnette* in Charleville-Mézières *.* Two key points should be noted: the importance of the collections of Alexandre Martineau de Soleinne (1784–1842) who, when he died, left a library of 50,000 books and documents on the universal history of the theatre. Within this exceptional collection, a large part of which is held in the *Bibliothèque nationale de France*, are to be found the manuscripts of about 70 comedies for puppets which were performed in the 18 ^th^ century in the theatres of the Foires Saint-Germain and Saint-Laurent in Paris. This is truly an extraordinary collection because in other European countries it is difficult to find the manuscripts of plays before 1850. The other important phenomenon is the high number of texts commissioned over the past twenty years from authors by companies of puppeteers, this is the result of the convergence of several public initiatives: institutional support for writing for the theatre (such as grants, residences, or prizes) meetings between authors and puppeteers organized by the *Centre National des Écritures du Spectacle* in Villeneuve-lès-Avignon at the beginning of the years 2000 ^3^, invitations sent to several writers by the *École Nationale Supérieure des Arts de la Marionnette* in Charleville-Mézières to come and work with the student ^4^.

In these three countries -Italy, Germany and France- it is therefore possible to find, with no exaggeration, hundreds of published texts and thousands of unpublished ones: outline sketches, farces, comedies, contemporary texts, opera librettos, dramas and tragedies from all periods, and of all dimensions from a half-page sketch to an epic cycle in 120 episodes of the Paladins of France, as played by the *Opera dei Pupi* in Sicily. But the situation is completely different in the rest of Western Europe, from Great Britain to Spain, and from Portugal to the Netherlands, where only a few dozen texts can be assembled, although the activity of puppeteers is documented from the 16 ^th^ century onwards. The rare authors whose texts have survived remain isolated cases, like that of Antonio José da Silva, the most important Portuguese dramatic author of the 18 ^th^ century, whose comedies were all written for the great cork puppets of the *Teatro do Bairo Alto* in Lisbon. From the abundant Spanish production of the Golden Century, to the comedies performed in London in the 18th Century, almost nothing remains, very few collectors have sought to assemble the texts from the popular tradition, very few have been published (except of course the different versions of the *Punch and Judy Show* in England), and museums have never developed a systematic and ambitious policy to save the puppet theatre heritage.

### Comparative approaches

The *PuppetPlays* project is therefore faced with a double problem: on the one hand, the over-abundance of texts in certain linguistic zones, and on the other, the very small number in most of the others. If this imbalance is too great to be corrected by simply selecting the resources thus described and analyzed, we need to have recourse to different strategies so that the research we are carrying out can produce significant results on the international level. One of these strategies involves the precise localization of the resources that have been assembled: the interactive geographical map linked to the data base for example does not indicate the place where the documents are held, but where the plays were written and performed. We can thus clearly identify, at the regional level, where the different puppet theatre traditions developed, and where the main centres of activity were.

Another strategy which is in the process of elaboration is the creation of educational pathways which will highlight the dynamics of the dissemination and transformation of a character, a narrative sequence, or an animation technique across different countries, languages and audiences. Thanks to its popular base and to the movement from place to place of its artists, the puppet theatre greatly contributed to the dissemination of common cultural references in Europe: legends, myths, Shakespeare plays, Jules Verne novels, melodramas, operas or tales, to name but a few.

Comparing the different versions of the same story makes it possible to link cultural zones where documentation concerning the presence of puppeteers is patchy, and to verify the superficial analyses which too often have remained centred on the local characters and their supposed specificity. From my point of view, it is necessary to deconstruct the identity mythologies in which many studies of the puppet theatre have remained confined: obsessed by the idea of the psychological and even biological cohesion of a character, the specialists of regional cultures have too often elevated the protagonists of the puppet theatre to “sites of memory” (in the sense given to the expression by Pierre Nora ^5^) for local identities, failing to mention the dynamics of circulation, borrowings and successive re-workings which have given them their vitality.

To give just one example: I am not convinced by the attempts to link the Pulcinella of the comedies for actors in the 18 ^th^ and 19 ^th^ centuries (the “ *pulcinellate*”) to the shows for Neapolitan glove puppets (the “ *guarattelle*”) as if they staged the same unique and coherent character with close links to the city of Naples, or to its immediate surroundings.

If we examine the iconographic traces they have left, we discover many similarities between the Pulcinella of the Italian puppet theatres (Neapolitan, but also Roman, Venetian, *etc.)* and the other European glove puppet traditions, and we can formulate the hypothesis that a common pool of “routines” (the fight against an animal, throwing the baby out of the puppet booth, the hanging of the executioner) which spread from Italy to Great Britain *via* France pre-existed the establishment in the local environment and the process of differentiation which led to the creation of the figures of Punch, Polichinelle and Pulcinella ^6^. It is only superficially that we can link the Pulcinella of the *guarattelle* to that of the actors’ theatre: it is more likely that the itinerant puppeteer clothed his protagonist, the puppet he held in his right hand which triumphed over all its enemies, in the attributes of the theatrical character most loved by the local audience.

## A specific entertainment or simply theatre?

### The theatre of the popular classes

This leads us to the question of the place of the puppet theatre within the wider context of the arts of the stage. Rather than considering the use of glove, string, and rod puppets as well as shadow figures or objects as forming a specific branch of the performing arts, the *PuppetPlays* project proposes to examine these productions as a theatrical form in the full sense, which means examining them with the critical instruments used for theatre studies, confronting them to the general history of playwriting and including them in the wider panorama of the culture of their times. If, thanks to the research of so many specialists, and to the memoirs published by so many artists, we today have a reasonably precise picture of the work of the puppeteers of the 18 ^th^ and 19 ^th^ centuries, too often this image remains fragmented and insular, cut off from any links with the surrounding theatrical landscape. Today it is necessary to include puppet theatre in the field of theatre itself, in order to highlight its specificities when they exist: specificities which do not always remain identical, and which do not have the same focus depending on the period, the cultural zones and social groups.

It is important to underline this last aspect: the puppet theatre is not a specific artistic domain with a fixed identity which has been consolidated over the centuries and within different cultures, but a set of variable practices, more or less distant from other theatrical forms, and more or less porous to their influence depending on the contexts in which they develop. Only by paying careful attention to puppet theatre repertoires will we be able to measure the distance from other theatrical forms and their porosity with regard to the actors’ theatre, but also in comparison with opera, pantomime, ballet, music hall, or later with cinema and television.

Even as far as popular shows are concerned, a detailed analysis of the texts should be the indispensable preamble to all research. How many studies only list the titles of the plays in the puppeteers’ repertoire without indicating what lies behind each one of these titles. Is it an original work? A faithful revival of an actors’ play? A reduction? A parody? a rewriting? Too often the intertextual approach only states that the puppet play is an “adaptation” of a stage success with the addition of a local comic character in the role of the servant. This makes it impossible to account for the strong dynamic involved in adaptation, or for the differentiation between “high” and “low” culture. Investigation should go beyond these superficial observations.

For example, the success of the character of Don Juan on puppet stages in Germanic countries at the end of the 18 ^th^ century and during the 19 ^th^ century involved a profound reworking of the plot: the protagonist no longer appears as the seducer of “ *mille e tre*” victims, his amorous adventures disappear from the dramatic action and the female characters are almost completely erased. It is because he is an unnatural son that Don Juan transgresses social norms and is punished: he is a son who insults his father and, in some versions, kills him because the latter refuses to pay his debts. The violence of the libertine’s revolt thus culminates in the crime that the 18 ^th^ century considered to be the most heinous of all: parricide. Whereas Don Juan is transformed into a hardened Prodigal Son, incapable of repenting, his servant Hanswurst or Kasperl becomes the popular hero and the representative of common sense with whom the audience can identify ^7^.

### The theatre of experimentation and of the avant-garde

However in many contexts, and contrary to what is generally thought, the repertoire of puppet theatres is not made up of adaptations of successes from the actors’ theatre, either because it follows its own traditions, feeding on stories handed down from generation to generation, and which are still in demand (for example, for string puppets, the legends of Faust, Genevieve of Brabant, Robert the Devil or the Temptations of Saint Anthony), or because from the 19 ^th^ century onwards the expressive possibilities of puppets have become the departure point for innovations and dramaturgical experimentation heralded by artists from the Symbolist and Modernist movements and the avant-gardes.

Indeed in these literary and artistic circles, the puppet theatre has become the laboratory for the stage of the future. Its reduced dimensions, the possibility of better controlling the stage action as a plastic event, the greater dramaturgical freedom which makes it possible to explore the poetic, the surreal, or the illogical, all exercise a fascination proportional to the burden of habit and timidity which afflict the big theatres. The beginning of the 20 ^th^ century marks a reversal in the history of the puppet theatre. It is no longer seen as an alternative or a substitute for the actors’ theatre, but a broadening of what theatre can be.

This is verified in the dramaturgies in which actors and puppets appear together in the same stage space. If already on the stage of the baroque theatre, the process of “ *mise en abyme*”, that is theatre within theatre, could introduce rod or glove puppets into comedies for actors (for example in Ben Jonson’s *Bartholomew Fair),* this phenomenon which regained in vitality at the beginning of the 20 ^th^ century with what Gino Gori called the “theatre of the grotesque”, has taken on a completely different dimension in the course of the last few decades. The confrontation between different types of stage presence no longer signifies a criticism of the “puppet-like” character (artificial, stereotypical) of human behaviour as was the case in the work of Massimo Bontempelli or Enrico Cavacchioli, but rather the de-centering of the human being within the framework of the performance. Puppets can represent animals, states of matter, natural phenomena, and abstract ideas. They can transform the stage into a poetic space where fictional beings appear as metaphors, metonymies, or allegories of other realities. I am thinking for example of Jean Cagnard’s text *Les Gens légers ^8^,* in which the character Petit Tas de Cendres (Little Pile of Ash) figures the victims of the Shoah who were killed in the gas chambers of the Nazi extermination camps. When we think today about what a post-anthropomorphic theatre might be like, I am convinced that we should look at the possibilities offered by the puppet theatre.

## Rethinking the historiography of Western theatre

### Reconsidering neglected genres and successful shows

Because the puppet theatre cannot be dissociated from the general landscape of the performing arts or, more precisely, from what Giovanni Moretti called the “theatrical system” of a period ^9^, thinking about its dramaturgy and its repertoires encourages us to look anew, and without prejudice, at the history of the theatre. Although for several decades researchers have begun to focus attention on provincial theatres, salon theatres, amateur companies, and popular entertainments, thus widening the spectrum traditionally covered by the history of the theatre, the *PuppetPlays* project takes into consideration areas of theatrical production where critical and historiographical criticism is practically inexistent. In particular, the study of 18 ^th^ and 19 ^th^ century rod and string puppet theatres whose programmes were very close to those of the actors’ theatres, has enabled us to rediscover neglected theatrical genres: for instance, military dramas, *féeries*, news and end of year reviews, and the histories of saints or bandits. Although these genres and sub-genres occupied an important place in the theatrical life of their times, it is difficult for them to find a place in the historiography of theatre which in many ways today remains structured by the succession of literary movements, and which excludes “minor” dramaturgical models.

One of the great methodological difficulties that we have encountered is the integration of puppet theatre into the general history of the stage. Indeed, our research, which relies heavily on the study of the collections of hand-written plays, does not only bring to mind forgotten genres, it also brings to light an indirect image of ordinary theatrical production, of day to day life on the great and small stages: anonymous plays, four-handed comedies, commercial successes lasting a few months or a few years which puppeteers take as a model or reproduce faithfully, but which theatre historians keep no record of. Their successes in fact came from reproducing tried and tested, even worn out, formulas, whereas the way the history of the art is written tends to place value on innovation, the break with, or at least the distance from, the expectations of the public. Our study is therefore hindered by the absence of critical and historiographical instruments which would enable us to examine with greater precision the repertoire of puppet theatres in relation to the context of production.

This reminds us that even a partial analysis of an unexplored documentary deposit like, in our case, the collections of puppet texts, forces us to reconsider the whole field to which it belongs. We find ourselves in the position of the archeologist who seeks to interpret, with the help of historians, the material brought to the surface by excavations, but who cannot find in their publications the information that he or she needs to understand what has been discovered. To produce the greatest number of results, the interpretation of a deposit needs to be set against other deposits. It should be possible to compare the texts played by puppeteers with those of the companies of actors, but not selected, not filtered by editors or historians, just as they were kept in the collections of museums and libraries, or the archives of theatres or indeed of the censor.

### The asynchrony of theatre practices

The archeology of theatrical repertoires seems to me to have become indispensable today to refocus our attention on the art of theatre as performance in order to continue to broaden the map of its manifestations, and to better understand what its cultural, political and social functions were at any given period.

Even if there remains a year and a half before the end of the *PuppetPlays* project, it is time now to sketch out our first conclusions. One of these conclusions is the confirmation of the limited heuristic power of the Hegelian historiographical model: that of a linear progression punctuated by successive leaps (the famous *Aufhebung* of the German philosopher) making it impossible to turn back – a model to which the success of “post dramatic theatre ^10^” theorized by Hans-Thies Lehmann has given a new lease of life. It is surprising to see that an explanatory schema, which today has been abandoned for the study of the history of human societies (we should remember that its last avatar was the hypothesis of the “end of history” formulated by Francis Fukuyama at the beginning of the 1990s ^11^) should still be used in the field of the history of the theatre.

In the same way that, in a particular social group, heterogeneous kinds of behaviour and modes of thought, diversely rooted in the past, co-exist, the “theatrical system” of a period is made up of simultaneous but not synchronized performance practices which are born, develop with varying degrees of intensity, and disappear or survive for different lengths of time. Certain literary registers or dramatic genres which have been abandoned by the actors’ theatre remain active on the puppet stage. This is true of the farce which finds its extension in the plays for Pulcinella, Polichinelle, Punch and many others. At the end of the 19 ^th^ century, it was generally thought that religious dramas (episodes from the Old Testament, the Nativity, the Passion, hagiographic legends) found in puppet plays (whether destined for a popular audience of for artistic and literary circles) the perfect place for the expression of religious sentiments. *Féeries,* melodramas and mime shows continued to be played by puppets in the first decades of the 20 ^th^ century – at the same time as poets and modernist artists or avant-gardists were exploring the expressive possibilities of this theatrical instrument to lay the basis for what we today call “visual dramaturgy”.

### Heterochrony of the theatrical text

But the investigation into the repertoires of puppet theatres does not only show the multiplicity of active temporalities simultaneously at work in any theatrical system, it also reveals how much the dramaturgy of this kind of theatre is made up of different historical strata, often within a single text. Because the puppet theatre brings closely together the *logos* and the *opsis*, the word and the image, it would seem that it rests, more so than the actors’ theatre, on the characteristic heterochrony of the visual arts highlighted by Aby Warburg, and theorized by Didi-Huberman in his essay *L’Image survivante ^12^*

These layers of time, perceptible in the dramaturgy of the puppet theatre, can be seen already in the impossibility of precisely dating certain works, or attributing them to only one author. Particularly in the domain of popular theatrical practices, the intergenerational transmission of texts, with additions, reworkings, and subsequent cuts leads us to reconsider the historiographic categories of origin and authorship.

Take for example the manuscript of a “ *féerie*”, *Le Voyage de Guignol dans la Lune* in the collections of the Musée des Arts de la Marionnette – Musées Gadagne in Lyons. The cover of the booklet gives the date of 1852-1854 and the name of the puppeteer, Louis Josserand the Elder, the son-in-law of the creator of Guignol, Laurent Mourguet. However, a series of indications inside the booklet reveals that in reality this is a copy produced in 1905 by another puppeteer, Joanny Durafour, and that several additions had been made including a scene invented by Louis Josserand’s son, several dialogues in verse introduced in 1889 for performances in Marseilles, and even a mime show added in 1903 by Durafour himself imitating a sequence from George Méliès’s film *Le Voyage dans la Lune* (1902). The text that we can read in this booklet is therefore the result of a process of rewriting over the course of half a century during which four puppeteers in turn contributed.

It is not only hand-written copies of puppet plays in the form of booklets handed down and recopied by several generations which bear witness through their additions and reworkings to the heterochronic dimension of these repertoires. The stage action itself may retain traces, now illegible, of ancient modes of thought, of customs or beliefs which have been long abandoned.

I will give just one example, that of different performances of the Temptation of Saint Anthony. This was a classic show, put on by itinerant puppet theatres between the 18 ^th^ century and the First World War. All the versions of this play, derived from popular traditions, which have come down to us have just one source: a *pot-pourri* of little songs written by Michel-Jean Sedaine around 1750 and published, often without any indication of the author, during the second half of the 18 ^th^ century. The original *pot-pourri* was rather erotic and satirical in inspiration (some editions were illustrated with “gallant”, as they were termed, etchings, whereas the puppet play was presented as a serious and moving hagiographic drama. The puppeteers therefore had to modify certain details or certain lines of the text, to erase the pornographic and anti-clerical aspects in order to adapt it to its new function as an edifying story for a popular audience. They also added the apotheosis of the saint, borne heavenward by angels, while in the *pot-pourri* Saint Anthony sang of his relief on seeing the demons flee. Trapped in the arms of Proserpina, he was on the point of making her husband, the Devil himself, cuckold.

The role which underwent the most modification was that of the little pig, the companion of the hermit in popular iconography. Whereas in the Sedaine version, the demons tormenting Anthony disguised the pig as a monk, clothing it in a hair shirt, their mistreatment changed radically on the puppet stage. In some versions, the end of a bellows was inserted into the pig’s backside, thus bringing to mind the medieval French *Soufflacul*; in another version, a piece of flaming oakum rope or a lighted firework was attached to its tail, and sometimes this was a live pig and not a puppet. Claudine Fabre-Vassas, an anthropologist who has studied the links between Saint Anthony’s pig and the cult of the dead in European folk traditions ^13^, has been able to collect some local legends from the Mediterranean Basin which recount how Anthony and his pig, Prometheus-like, descended into the depths of Hell to steal fire and offer it to humans – a fire which then remained attached to the pig’s tail. We can therefore advance the hypothesis that in their plays puppeteers recycled traces of forgotten symbolic systems, images detached from their original contexts and chosen for their effectiveness on stage. Developed progressively by amalgamating scattered fragments, deflected and deformed to conform to new expressive strategies, the dramaturgy of the popular puppet theatre is, like the images referred to by Didi-Huberman, inhabited by many ghosts, asynchronic memories which remind us that the theatrical performance may not only be a heterotopia, in the sense that Michel Foucault gave to the word, but also a heterochrony, an alternative construction to social time.

These reflexions are an invitation to examine with greater attention the dramaturgy of the puppet theatre. For too long these texts have been neglected, either because they documented an art which was considered to be “minor”, or because the fascination exercised by the theatrical object, the puppet – this reduction of a living being – focalized everyone’s attention.

When the person in charge of the *Sammlung Puppentheater* in the *Stadtmuseum* in Munich heard about the *PuppetPlays* project, she said that she hoped that our research would awaken their “Sleeping Beauty”, the enormous collection of published texts and manuscripts held in their document centre, that no one consulted and that they did not know what to do with, unless it was used to identify the characters represented by the puppets exhibited in the museum showcases.

Not only are we “awakening” the collections in the different conservation sites that we are exploring, but by describing and referencing these resources in our data base, we aim to give life to this forgotten patrimony. Many questions can be examined in the light of puppet theatre repertoires. Some concern the history of the theatre, and as I have tried to show, they invite us to reconsider the way we recount this history. Other questions concern theatrical poetics and the possibility of identifying the dramaturgical specificities of writing for the puppet stage. The documentary deposits that we have identified and that we are exploring, the resources that we describe in the data base and those that, in an online anthology, we place at the disposal of users, and the analyses that we present in our scientific publications - all this material as well as our collections, methodology, and results aim to nourish the research in other disciplinary fields: linguistics, literary studies, sociology, cultural studies, and anthropology… As a global phenomenon, the puppet theatre stands at the crossroads of many different disciplines in the humanities. We hope therefore that by giving greater visibility to its repertoire the *PuppetPlays* project will contribute to the recognition of the puppet theatre as a crucial part of European cultural patrimony and the contemporary artistic landscape.

## Ethics and consent

Ethical approval and consent were not required.

## Data Availability

No data are associated with this article.

## Footnotes

^1^ Steve Tillis, *Towards an Aesthetics of the Puppet: Puppetry as a Theatrical Art.* New York and London: Greenwood, 1992.

^2^ Michèle Anne De Mey, Jaco van Dormael, *Kiss & Cry* (2011); *Cold Blood* (2015).

^3^ *La Chartreuse 1973–2013, Le monument aux écritures*. Montpellier : L’Entretemps, 2013, p. 185–187.

^4^ Lucile Bodson, Margareta Niculescu, Patrick Pezin (ed.), *Passeurs et complices / Passing it on. Institut International de la marionnette, École nationale supérieure des arts de la marionnette*. Montpellier: L’Entretemps, 2009.

^5^ Pierre Nora (ed.), *Les Lieux de mémoire*. Paris: Gallimard, 1984-1992.

^6^ *Polichinelle, entre le rire et la mort. Filiations, ruptures et régénération d’une figure traditionnelle*. « Les Rencontres du Musée Gadagne ». Milano: Silvana editoriale, 2015.

^7^ Ved. Jean Boutan, « De Don Juan à Kasperl : le devenir populaire d’un mythe européen dans le théâtre de marionnettes en Europe centrale », forthcoming publication in the *Revue de Littérature comparée*.

^8^ Jean Cagnard, *Les Gens légers*, Montpellier: Espaces 34, 2006. See also D. Plassard, Carole Guidicelli, « Haunted Figures, Haunting Figures: Puppets and Marionettes as Testimonies of Liminal States », *Skenè*, vol. 8, n° 1 ( *Puppet, Death, and the Devil: Presences of Afterlife in Puppet Theatre*), 2022, p. 11–33.

^9^ Giovanni Moretti, *Attori e barracche. Il Fornaretto nel sistema teatrale*. Torino : Seb27, 2002.

^10^ Hans-Thies Lehmann, *Post-dramatic Theatre*. London: Routledge, 2006.

^11^ Francis Fukuyama, *The End of History and the Last Man.* New-York: Free Press, 1992.

^12^ Georges Didi-Huberman, *The Surviving Image*. University Park, Pennsylvania: Penn State University Press, 2016.

^13^ Claudine Fabre-Vasses, « Du cochon pour les morts ». In: *Études rurales*, n° 105–106 (Retour des morts), 1987, p. 181–212.

