# Supplementary material for: The ERC  PuppetPlays project : contribution for a non-linear history of the European theatre
Source: Open Res Eur. 2023 May 2;3:72. [Version 1] doi: 10.12688/openreseurope.15807.1 (PMC10446019; doi:10.12688/openreseurope.15807.1)
Supplement: Peer review history

# Peer review: Reviewer response for version 1

**DOI:** 10.21956/openreseurope.17077.r31420

**Published:** 2023-06-30

**Authors:** Stefano Locatelli

**Affiliations:** 1 Department of History, Anthropology, Religions, Art and Performing Arts, Universita degli Studi di Roma La Sapienza, Rome, Lazio, Italy

The article is a presentation of the ERC Advanced Grant Project *PuppetPlays. Reappraising Western European Repertoires for Puppet and Marionette Theatres*, directed by Didier Plassard.

 The main object of the project is the repertoire of text written for puppet theatre from 17 ^th^ century in Europe.

 The author, in order to include the phenomenon of the puppet theatre into the general sphere of theatricality, uses the category of "double vision" theorized by Steve Tillis.

 The aim of the project is not only to analyze puppet theatre as sort of "minor" theatre, or substitute of theatre, in contexts where theatre was absent (for economic, moral or geographical reasons).

 The project, in fact, traces more interesting hypothesis of research in the field of co-existence of actors theatre and puppet theatre, focusing on the **" *expressive potential"*** of puppet theatre.

 The research is, above all, dramaturgical.

 The project explains in detail a first preliminary and quantitative objective: mapping the dramaturgical repertoire of puppet theatre in Western Europe. The construction of a database (puppetplays.eu) is clearly defined, also evidencing (with some precise examples) the difficulties and disparities, in different countries, due to differences in conservation, typology of authoriality, centralization or dissemination of collections, quantity of published or unpublished texts. The approach of the database itself is comparative, aiming to provide a tool capable of highlighting how puppet theatre contributed to the dissemination of common cultural references in Europe.

 The importance of the project is highlighted in the last part of the open letter. The author dwells on the methodological difficulties encountered in integrating puppet theatre with the general history of theatre.

 Of particular relevance and heuristic potential is the hypothesis of applying to dramaturgy of the puppet theatre the category of heterochrony of the visual arts highlighted by Aby Warburg and more recently theorized by Didi-Huberman. 

 The analysis of the puppet theatre and its expressive potential, not as a "minor" theatre or surrogate theatre, but as an integral part of the history of the theatre, defines the project as extremely important, hypothesizing the possibility of a study which, also thanks to the quantitative approach, will allow a "in vitro" study of some very relevant dynamics for the entire history of western theatre in the modern age and its role in the construction of social time.

 The open letter describes the project more than adequately from a scientific point of view in all its components.

Where applicable, are recommendations and next steps explained clearly for others to follow? (Please consider whether others in the research community would be able to implement guidelines or recommendations and/or constructively engage in the debate)

Yes

Does the article adequately reference differing views and opinions?

Yes

Are all factual statements correct, and are statements and arguments made adequately supported by citations?

Yes

Is the rationale for the Open Letter provided in sufficient detail? (Please consider whether existing challenges in the field are outlined clearly and whether the purpose of the letter is explained)

Yes

Is the Open Letter written in accessible language? (Please consider whether all subject-specific terms, concepts and abbreviations are explained)

Yes

Reviewer Expertise:

Modern Theatre, with particular reference to XVIIIth Century; Italian Theatre.

I confirm that I have read this submission and believe that I have an appropriate level of expertise to confirm that it is of an acceptable scientific standard.

# Peer review: Reviewer response for version 1

**DOI:** 10.21956/openreseurope.17077.r31423

**Published:** 2023-06-30

**Authors:** Nancy Delhalle

**Affiliations:** 1 Universite de Liege, Liège, Walloon Region, Belgium

In this text, Didier Plassard reports, in a very precise way on the scientific level and very stimulating on the intellectual level, the research he conducts with his team on the dramaturgy of puppet theatre. The article presents the challenges of this research, supported by European funds, but also its constraints, the perspectives it opens up and its potential contribution to science, in this case to the history of theatre.

 If puppet theatre is still relatively unknown, it is because it has often been considered a substitute for actor theatre. However, as Plassard points out, puppet theatre is omnipresent socially, both in “popular” circles and in the elite world. The research therefore tends to demonstrate the impossibility of validating a difference between high and low culture.

 Working on the texts written for this theatre allows to have a material that crosses time and does not need to be reconstituted before being studied. In this perspective, research can be shared both in scientific circles and among theatre lovers and students. This guarantee of dissemination of knowledge is reinforced by the choice to make the results of the research, but also the texts themselves, in the form of a database which will allow a very wide access geographically and socially.

 The article is relevant to the difficulties of the enterprise and the methodological responses to obtain valid results at European level. For example, the localization of texts requires researchers to build networks with amateurs and collectors and, on a other side, chronological fluctuations need to be contextualized. This methodological construction in the form of work in progress allows Didier Plassard to identify some well-founded research hypotheses. Thus, the comparison of different versions of the same story allows to show the diffusion in Europe of cultural references and thus to overcome a regionalist approach that often maintains the puppet theatre in the status of a « place of memory » (Nora) for a local identity. This is a very fruitful thesis for cultural history and theatre history and, specifically, for a history of European theatre.

 Another observation makes it possible to renew the historical approach of the theatre: the puppet theatre often uses existing formulas where the history of the theatre favours innovation and distance from the expectations of the public.

 In the end, Didier Plassard’s article creates real research perspectives that take theatre into account both as art and as a social practice. This articulation is too rare in the field of theatrical studies that often compartmentalizes the aesthetic approach of works and the study of social animation practices by the theatre. We can therefore only encourage a wide dissemination of this research, which is still ongoing, which combines issues of scientific excellence and popularization

Where applicable, are recommendations and next steps explained clearly for others to follow? (Please consider whether others in the research community would be able to implement guidelines or recommendations and/or constructively engage in the debate)

Yes

Does the article adequately reference differing views and opinions?

Yes

Are all factual statements correct, and are statements and arguments made adequately supported by citations?

Yes

Is the rationale for the Open Letter provided in sufficient detail? (Please consider whether existing challenges in the field are outlined clearly and whether the purpose of the letter is explained)

Yes

Is the Open Letter written in accessible language? (Please consider whether all subject-specific terms, concepts and abbreviations are explained)

Yes

Reviewer Expertise:

Theatrical and performance studies

I confirm that I have read this submission and believe that I have an appropriate level of expertise to confirm that it is of an acceptable scientific standard.

# Peer review: Reviewer response for version 1

**DOI:** 10.21956/openreseurope.17077.r31421

**Published:** 2023-06-01

**Authors:** Tiffany Stern

**Affiliations:** 1 Shakespeare Institute, University of Birmingham, Birmingham, England, UK

This article describes the ERC project to collect and categorise repertories for puppet shows across Western Europe: a fascinating and much-needed venture. It defines its terms – in particular, what constitutes a puppet – and explains the varying nature of documents that survive from puppet performances. Originally, it set out to explore whether there were repertories unique to puppets, or whether puppets performed a version of actors’ theatre. The answer, however, differs according to place and time: puppet repertory sometimes reflects current theatre, sometimes earlier ‘lost’ theatre, sometimes bits of folklore and popular songs. This important project is expanding our sense of what constitutes theatre, cultural memory, genre and authorship. It brilliantly reminds us that theatre history often focuses on stage innovation, whereas puppet theatre often depends on tried and tested formulas and keeps alive performances and plays often abandoned by actor theatre. ‘Awakening’ underused collections of puppet texts and enabling them to be part of an expanded theatre narrative, is just one of the contributions made by this project and described in the letter.

 The project is being manifested in a database of puppet performances from, it says, the repertoires of puppet theatres in Great Britain, France, Spain, Portugal, Italy, Switzerland, Austria, Germany, Belgium, Luxembourg and the Netherlands (though notably, not all those countries have left much of a repertory, and not all of them are, as yet, in the database); and in a book. Both are needed, and both will change the way we understand the theatre of Western Europe.

 The rationale for the letter is clear and detailed, and the project is so fresh that differing views are not really an issue (though expansion of those views will be reflected upon below). Factual statements are correct, citation is clear, and for the most part the language is accessible or, if not immediately accessible, explained.

 What follows are my questions about the project itself, and about next steps.

**1. Selection criteria**

 The project has unearthed a staggering amount of material, so much so that it has had to be selective – of the projected 2000 puppet shows it once hoped to record (which would itself only have been a selection), it has now decided in favour of 1000, and has so far catalogued around 500. Given the amount of material, and the care with which it is being gathered and processed, this is no surprise. The process of selection, however, was not made explicitly clear in this article: how were the 1000 chosen, and will information be supplied about the texts that have not been picked? A mention is made of " *our own selection criteria (age, diversity, variations on the same story)*", but I would like to have understood better how the criteria was determined in the first place, as well as how it is being applied. An online anthology of 300 of the texts will also be put together: again, this will be hugely useful, and, again, I’d like to know how this particular 300 has been chosen. Will all countries be covered in the sample (which might give the misleading notion that texts survive in comparative numbers from all countries)? I’d also like to know more about the book’s form. Will the 300 be supplied in native languages and/or translated into English? Will they be ‘edited’ (will missing stage directions etc. be added)? Will there be notes?

**2. Date range**

 The date range was not explicitly addressed in the article, presumably because, as it points out, puppet texts are themselves difficult to date, and date of writing/publication can be far from date of origin. Nevertheless, it would have been useful to have some sense of period: for instance, whilst the 16 ^th^ century is mentioned, the earliest surviving text on the actual database is from the 17 ^th^ century, 1676, and indeed, currently there are only 3 puppet texts on that site that are from before 1800 – meaning that the selection in reality seems to be largely of 19 ^th^ and 20 ^th^ century plays.

**3. Country range**

 The project has revealed an enormous disparity of resources: Italy provides many, many puppet shows over several regions; Germany and France also leave a rich puppet show record. From Great Britain to Spain, and from Portugal to the Netherlands, however, only a few dozen texts can be assembled (checking the database as it currently stands reveals no puppet texts from those countries so far). The project cannot solve this disparity but could explain how it will handle it. Given the project’s perimeters, will whatever survives from e.g. the Netherlands be mounted on the site? That would mean that the process of selection that governs texts from France, Germany and Italy will not be used for the texts from these other countries. Is there something to be said for narrowing the range of countries in order to create a more focused narrative?

**4. Nature of analysis**

 The project examines literary register and theatrical technique in puppet shows and employs " *the critical instruments used for theatre studies*" to allow for comparison between puppet texts and opera, pantomime, ballet, music hall, or, later, cinema and television. This is very useful, but I wonder whether the method is in danger of dictating the findings – in that, it is likely to bring out similarities with other forms of theatrical performance. I’d be intrigued to know more about the texts that utilise what puppets can do that is unlike actor theatre. Puppets of all kinds can hit one another hard without causing pain and often do so disproportionately as a result. They can also easily fly and sink, lose and regain limbs etc. It would be helpful to hear more about the differences between puppets and actor theatre (even when performing the same texts). Also, given how often puppets also favour military dramas, histories of saints, stories of local bandits etc., I wonder whether a more explicit focus on non-theatre sources might also be useful. Those particular topics are often in or from ballads and chapbooks, and there might be mileage in looking for comparisons outside actor theatre altogether and in the alternative worlds of song and folk book.

**Next steps**

 It was not totally clear to me what would happen after the completion of the database and the book. I do have some thoughts, however. One is that the database should be made open to contributions from people not involved in the project itself. That would enable the website to make the most of what is known by academics, libraries, museums, puppeteers and interested others across Europe. I’m thinking in terms of sites like the Lost Plays Database, that ‘crowd source’ information.

 A second is that the database might expand to record, when it can, surviving puppets themselves. This article does not address the material theatre much – though surviving puppets, stages and props might indicate the content of some of the lost puppet shows. Given that puppet shows are not always primarily about written text (sometimes they are oral, their speakers illiterate), it would be helpful to have alternative ways of capturing lost shows. Moreover, while actors from the distant past are dead and gone, that is not true of all old puppets, some of which survive. They themselves are therefore another unique form of theatre history and would wonderfully add to this site.

 In conclusion, this is a thought-provoking letter describing a major project the findings of which are, and will continue to be, extraordinary. It draws attention to the fact that, at various historical times, both puppet and actor theatre co-exist in the same place – meaning that puppet theatre must be contributing something in its own right – and with its questions and astounding archival work, starts to think through what those contributions might be. My suggestions arise from my excitement at the project itself, and my thoughts about the greater possibilities to which it gives rise.

Where applicable, are recommendations and next steps explained clearly for others to follow? (Please consider whether others in the research community would be able to implement guidelines or recommendations and/or constructively engage in the debate)

Yes

Does the article adequately reference differing views and opinions?

Yes

Are all factual statements correct, and are statements and arguments made adequately supported by citations?

Yes

Is the rationale for the Open Letter provided in sufficient detail? (Please consider whether existing challenges in the field are outlined clearly and whether the purpose of the letter is explained)

Yes

Is the Open Letter written in accessible language? (Please consider whether all subject-specific terms, concepts and abbreviations are explained)

Yes

Reviewer Expertise:

Theatre and book history, 16th-18th century, including puppets

I confirm that I have read this submission and believe that I have an appropriate level of expertise to confirm that it is of an acceptable scientific standard.
